# Quantifying dietary acid load in U.S. cancer survivors: an exploratory study using NHANES data

**DOI:** 10.1186/s40795-022-00537-4

**Published:** 2022-05-03

**Authors:** Maximilian Andreas Storz, Alvaro Luis Ronco

**Affiliations:** 1 Department of Internal Medicine II, Center for Complementary Medicine, Faculty of Medicine, University of Freiburg, Freiburg, Germany; 2 Unit of Oncology and Radiotherapy, Pereira Rossell Women’s Hospital, Bvard. Artigas 1590, 11600 Montevideo, Uruguay; 3 School of Medicine, CLAEH University, Prado and Salt Lake, 20100 Maldonado, Uruguay; 4 Biomedical Sciences Center, University of Montevideo, Puntas de Santiago 1604, 11500 Montevideo, Uruguay

**Keywords:** Dietary acid load, Acidosis, Cancer, Survivorship, Nutrition, Epidemiology, Nutrient intake, Prevention

## Abstract

**Background:**

Diet is an important determinant of systemic pH and acid–base regulation. A frequent consumption of acid-inducing foods (including processed meats and cheese) combined with a low intake of base-inducing foods (such as fruits, legumes and vegetables) increases Dietary Acid Load (DAL), which has been associated with an increased risk for certain cancers. DAL also appears to be of paramount importance in cancer survivors, in whom it was associated with increased mortality and poor overall physical health. Literature on DAL in cancer survivors, however, is scarce and limited to a few studies.

**Methods:**

Using cross-sectional data from the National Health and Nutrition Examination Surveys (NHANES), we sought to quantify DAL in U.S. cancer survivors and contrasted the results to the general population. DAL was estimated using established formulas (Potential Renal Acid Load (PRAL) and Net Endogenous Acid Production (NEAP)).

**Results:**

Our study comprised 19,413 participants, of which 1444 were self-reported cancer survivors. Almost 63% of cancer survivors were female (weighted proportion) with a mean age of 61.75 (0.51) years. DAL scores were consistently higher in cancer survivors (as compared to the general population) after adjustment for confounders in multivariate regression models. These differences, however, were not statistically significant (*p* = 0.506 for NEAP_F_, 0.768 for PRAL_R_ and 0.468 for NEAP_R_, respectively). Notably, DAL scores were positive throughout (> 0 mEq/d) in cancer survivors, suggesting an acidifying diet. Specific examples include mean PRAL_R_ scores > 11 mEq/d in cancer survivors aged 55 years and mean NEAP_F_ scores > 50 mEq/d in cancer survivors aged 40–60 years).

**Conclusions:**

The acidifying diet in this sample of cancer survivors warrants caution and requires further investigation. Comparably high DAL scores have been associated with adverse health outcomes and an increased mortality in previous studies in breast cancer survivors. Thus, increased awareness as well as additional clinical trials in this field are urgently warranted.

## Background

Diet affects the human acid-base status [1], and may influence systemic pH, metabolism and acid–base homeostasis [2, 3]. Dietary acid load (DAL) is determined by the balance of base-inducing foods (such as fruits and vegetables) and acid-inducing foods (including meats, eggs, and cheese) [4, 5]. Plant foods are abundant in metabolizable organic anions and generally decrease DAL, with green leafy vegetables (celery, broccoli, spinach), raisins and berries having a particularly alkalinizing effect [5, 6]. In contrast, the oxidation of sulfur-containing amino acids (methionine, cysteine and homocysteine) found in meat and meat products generates sulfate and thereby increases DAL [7, 8].

A high DAL has been positively associated with insulin resistance and type-2-diabetes [4, 9], chronic kidney disease [10], and cardiovascular disorders [11] in numerous epidemiological studies.

It has been argued that diet-dependent acid load could also predispose individuals to an increased cancer risk [12, 13], given that chronic acid–base imbalances can affect cellular and molecular activities that lead to tissue inflammation and cell transformation, which both may stimulate carcinogenesis or tumor progression [13–16]. Keramati et al. recently performed a systematic review and meta-analysis exploring potential association of a high DAL and cancer risk [17]. The authors emphasized that a high DAL may lead to a decreased adiponectin secretion (which plays a pivotal role in the development and progression of multiple malignancies [18]) and to an elevation of cortisol production and circulating IGF-1 levels [17]. The latter is a potent stimulant of several signaling pathways through binding to its cell surface receptor and induces cancer cell proliferation, survival, and migration [17, 19]. The authors also suggest that an acidogenic diet may increase carcinogenesis by elevation of insulin resistance [17].

In fact, DAL has been associated with an increased risk for certain types of cancer in various epidemiological studies [12, 20–26]. Positive associations of a high DAL have been reported with regard to prostate [20], breast [12, 22], lung [23], colorectal [24, 25], and pancreatic cancer [21]. In their meta-analysis, Keramati et al. pooled 10 studies and found that individuals with the highest DAL scores had a 66% increased risk of cancer (compared to those with the lowest DAL, *p* < 0.001) [17].

Although the total number of available studies in this particular field is still limited, there is now accumulating evidence that DAL could be an important modifiable risk factor to prevent from certain cancers.

Of note, the majority of studies focused on DAL as a risk factor in healthy individuals, while few studies addressed the role of DAL in cancer survivors [27–31]. Data from the Women’s Healthy Eating and Living (WHEL) study suggested that a higher DAL increased total mortality and breast cancer-specific mortality in a cohort of 2950 early stage breast cancer survivors [27]. An increased DAL was also significantly associated with increased plasma C-reactive protein (CRP) and hemoglobin A1c (HbA1c) levels, and reduced overall physical health in breast cancer survivors [28, 29]. In light of this mounting evidence, several experts called for adding DAL scores to dietary guidelines for breast cancer survivors [27]. However, the data supporting this call is based on a single cohort of breast cancer survivors from the WHEL study.

Additional trials and investigations using data from other cohorts are urgently warranted to gain a better (and more detailed) understanding of DAL in cancer survivors. We sought to address this gap in the literature and investigated DAL in U.S. cancer survivors from the National Health and Nutrition Examination Surveys (NHANES). The main aims of our study were twofold: a) to quantify DAL in U.S. cancer survivors, and b) to compare the results to the general U.S. population.

## Methods

### Study design and population

Our investigation is based on cross-sectional, population-based data from the NHANES [32]. NHANES is a major program of the National Center for Health Statistics (NCHS) designed to assess the health and nutritional status of non-institutionalized adults and children in the United States of America. The NHANES program began in the early 1960s and examined approximately 5000 people annually in 15 different counties across the country since 1999 [33]. The NHANES is an ongoing program which has two major components: an interview and an examination component. The interview includes demographic, socioeconomic, dietary, and other health-related questions [32]. The examination component consists of physiological and medical measurements, as well as laboratory tests administered by specialized medical personnel. Detailed information on both components may be obtained from the NHANES website [32].

NHANES data has been frequently used to investigate cancer-related and health-related questions [34–40]. For our present study, we used data from multiple NHANES cycles (2007/2008, 2009/2010, 2011/2012, and 2013/2014) to increase the potential sample size for analyses stratified by population subgroups. NHANES was approved by the National Center for Health Statistics research ethics review board [41]. Written informed consent was obtained from all participants.

### Data collection

We used data from various NHANES modules, including demographic data, anthropometric data, dietary data and questionnaire data. Demographic data included age (in years), gender (female and male), race/ethnicity (Mexican American, Other Hispanic, Non-Hispanic White, Non-Hispanic Black, Other Race - Including Multi-Racial), marital status (married or living with a partner, widowed/divorced/separated, never married), education level (less than 9th grade, 9-11th grade, high school graduate/ general education diploma or equivalent, some college or associate degree, college graduate or above) and annual household income (under $20,000 or over $20,000).

Examination data comprised body weight, height and body mass index (BMI), which were obtained from the body measures dataset and treated as a continuous variable.

### Dietary data

Dietary data were obtained from the nutritional assessment component of the NHANES, which included a 24-hour dietary recall interview for all participants [42]. The main objective of this module was to obtain detailed dietary intake information from NHANES participants [43]. All dietary interviews were carried out in private rooms in the NHANES mobile examination centers and were conducted in person by specifically trained dietary interviewers fluent in both English and Spanish [42, 43]. The specifically equipped dietary interview room contained a standard set of measuring guides that were used to help the respondent report the volume and dimensions of the food items consumed. The dietary intake data were used to estimate the types (and amounts) of foods and beverages consumed during the 24-hour period prior to the interview (midnight to midnight). Based on these data, energy intake and nutrient intake were estimated.

The NHANES dietary interview component, called “What We Eat in America” is conducted as a partnership between the U.S. Department of Health and Human Services and the U.S. Department of Agriculture (USDA) [42]. All “What We Eat in America” data was collected using USDA’s dietary data collection instrument, called the Automated Multiple Pass Method [44]. The AMPM is a fully computerized recall method designed to provide accurate means of collecting intakes for large-scale national surveys [42, 44]. Additional information may be obtained from the NHANES website. The detailed dietary examination protocol and data collection methods are fully documented in the NHANES dietary interviewer’s procedure manuals [45].

Dietary data used for this study included daily energy intake (kcal/d), fiber intake (g/d), and intake of macronutrients (protein, fat and carbohydrate; all reported in g/d) and selected micronutrients necessary for the DAL estimations (calcium, magnesium, phosphorus and potassium; all reported in mg/d).

### Cancer status

Cancer survivorship status was self-reported and based on the question “Have you ever been told by a doctor or other health professional that you had cancer or a malignancy of any kind?” from the medical conditions section. To stratify analyses by cancer sites, we made use of the question “What kind of cancer was it?”, which was included in the same module. In a first model, we combined all cancer types regardless of their site, except for skin cancers (non-melanoma and unspecified skin cancers) which were not included in this model. Afterwards, we performed sub-analyses stratified by specific cancer sites, including prostate cancer and breast cancer. Breast and prostate cancer were chosen in light of their aforementioned associations with DAL [20, 22, 27–30], and because of the modest case numbers (at least 60 cases for each cancer type) per cycle.

### Dietary acid load estimations

We described the employed methods for the calculation of DAL in our previous publications in detail [5, 22]. In brief, we used the formulas developed by Remer & Manz and Frassetto et al. to calculate Net Endogenous Acid Production (NEAP) [2] and Potential Renal Acid Load (PRAL) from diet [6, 46, 47]. PRAL_R_ (in mEq/d) was calculated according to the modified Remer formula:

$${\mathrm{PRAL}}_{\mathrm{R}}\left(\mathrm{mEq}/\mathrm{day}\right)=\left(0.49\ast \mathrm{total}\ \mathrm{protein}\ \mathrm{intake}\right)+\left(0.037\ast \mathrm{phosphorus}\ \mathrm{intake}\right)-\left(0.021\ast \mathrm{potassium}\ \mathrm{intake}\right)-\left(0.026\ast \mathrm{magnesium}\ \mathrm{intake}\right)-\left(0.013\ast \mathrm{calcium}\ \mathrm{intake}\right).$$

Net endogenous acid production was estimated based on Remer’s formula (NEAP_R_) and included average intestinal absorption rates of ingested protein and micronutrients (PRAL_R_) and anthropometry-based estimates for organic acid excretion (OAest) [6]:

$${\mathrm{Estimated}\ \mathrm{NEAP}}_{\mathrm{R}}\left(\mathrm{mEq}/\mathrm{d}\right)=\mathrm{PRAL}\ \left(\mathrm{mEq}/\mathrm{d}\right)+\mathrm{OAest}\ \left(\mathrm{mEq}/\mathrm{d}\right)$$

OAest (mEq/d) was calculated as follows:

$$\mathrm{Individual}\ \mathrm{body}\ \mathrm{surface}\ \mathrm{area}\times 41/1.73$$

Body surface area was calculated with the formula of Du Bois and Du Bois:

$$\mathrm{Body}\ \mathrm{surface}\ \mathrm{area}\ \left({\mathrm{m}}^2\right)=\left(0.007184\times \mathrm{height}\ {\left(\mathrm{cm}\right)}^{0.725}\times \mathrm{weight}\ {\left(\mathrm{kg}\right)}^{0.425}\right)$$

NEAP_F_ was estimated based on the formula by Frassetto et al., which considers daily total protein intake and potassium intake.

$${\mathrm{NEAP}}_{\mathrm{F}}=\left(\mathrm{mEq}/\mathrm{d}\right)=\left(54.4\times \mathrm{protein}\ \left(\mathrm{g}/\mathrm{d}\right)/\mathrm{potassium}\ \left(\mathrm{mEq}/\mathrm{d}\right)\right)-10.2$$

The reader is referred to the work of Parmenter et al. for additional background information on all three employed formulas [48, 49]. Negative DAL scores (PRAL_R_ < 0 mEq/d) indicate an alkaline-forming potential, whereas positive scores (PRAL_R_ > 0 mEq/d) indicate an acid-forming potential.

### Statistics

We used STATA 14 statistical software (StataCorp. 2015. Stata Statistical Software: Release 14. College Station, TX: StataCorp LP) for our statistical analysis. Stata survey commands were used to account for the NHANES survey design characteristics and population weights. Based on the NHANES guidelines, we generated an 8-year weight (2007–2014) to obtain weighted percentages adjusted to the US adult population. We compared all aforementioned variables between self-identified cancer survivors and the general population (who denied a previous diagnosis of cancer). Participants were not matched for age, sex, ethnicity, cancer status or any other variable. For this study, only participants with a full dataset were considered in the final analysis.

Continuous variables were described with their mean and standard error in parenthesis (if normally-distributed) or median and interquartile range in parenthesis (when not-normally distributed). For categorical variables we reported the number of observations (n) as well as the weighted proportions in parenthesis. Reliability of estimated proportions was assessed based on the 2017 NCHS guidelines [50], which consider the Korn–Graubard confidence interval (CI), CI widths, sample size, and degrees of freedom to assess reliability of a proportion and to determine whether it can be presented [51]. For this step, we also made use of the user-written post-estimation command “kg_nchs” in Stata [51]. Unreliable proportions, that is proportions that did not met the NCHS standards were clearly marked.

Continuous and normally distributed variables were compared between cancer survivors and the general population using two-sample Student’s t-tests. We assessed potential associations between cancer survivorship status and categorical variables using STATA’s design-adjusted Rao-Scott test. Moreover, we conducted a series of linear regression analyses to examine the relationship between all 3 DAL scores (NEAP_F_, PRAL_R_, NEAP_R_) and a selected set of independent variables. Predictor variables were chosen based on previous studies in the field and included age, gender, race/ethnicity, body mass index, total energy intake and cancer status [52–54].

Adjustment for total energy intake was necessary because cancer survivors were, on average, significantly older and had a lower total energy intake as compared to the general population. Only candidate predictors of interest and a bivariate relationship of significance (*p* < 0.25) with the response variables (DAL scores) were included in the multivariate logistic models. Multivariate linear regression models were constructed for all cancer sites combined, and for breast and prostate cancer, respectively. Margin plots were used to display marginal predicted values of NEAP_F_, PRAL_R_ and NEAP_R_ stratified by cancer survivorship status at all possible increments of 5 units in age. Statistical significance was determined at α = 0.05 and all employed tests for statistical significance were two-sided.

## Results

The total sample for this study comprised 19,413 participants. Our sample included 1444 self-reported cancer survivors. The remaining 17,696 participants denied a diagnosis of cancer in the past.

Cancer survivors had a mean age of 61.75 years and almost 63% (weighted proportion) were female (Table 1). More than 80% of cancer survivors were of Non-Hispanic White origin, and almost 2/3 were married or lived with a partner. Mean BMI was slightly higher in cancer survivors (29.20 kg/m^2^) as compared to the general population (28.85 kg/m^2^), however, this difference was not statistically significant (*p* = 0.210). Table 1 shows other demographic data characterizing our sample of cancer survivors.

**Table 1:** Demographic, anthropometric and clinical characteristics – a comparison between cancer survivors and the general population

	General population *n* = 17,969	Cancer Survivors: *n* = 1444	*p* ^a^
Gender			**< 0.001**
Male	*n* = 8783 (48.66)	*n* = 632 (37.21) ^c^	
Female	*n* = 9186 (51.34)	*n* = 812 (62.79) ^c^	
Ethnicity			**< 0.001**
Mexican American	*n* = 2781 (8.75)	*n* = 106 (3.04) ^c^	
Other Hispanic	*n* = 1800 (5.48)	*n* = 96 (2.80) ^c^	
Non-Hispanic White	*n* = 7730 (66.94)	*n* = 911 (83.35) ^c^	
Non-Hispanic Black	*n* = 3918 (11.76)	*n* = 266 (7.40) ^c^	
Other Race - Including Multi-Racial	*n* = 1740 (7.06)	*n* = 65 (3.22) ^c^	
Marital Status			**< 0.001**
Married/living with a partner	*n* = 10,570 (62.97)	*n* = 870 (65.19)	
Widowed/Divorced/Separated	*n* = 3842 (17.28)	*n* = 477 (28.59) ^c^	
Never married	*n* = 3557 (19.74)	*n* = 97 (6.21) ^c^	
Annual household income			**0.586**
Under $20,000	*n* = 4000 (15.05)	*n* = 354 (15.69)	
Over $20,000	*n* = 13,969 (84.95)	*n* = 1090 (84.31)	
Education level			**0.144**
Less than 9th grade	*n* = 1799 (5.29)	*n* = 152 (5.04)	
9-11th grade ^b^	*n* = 2756 (11.82)	*n* = 216 (10.78)	
High school graduate/GED or equivalent	*n* = 4136 (22.68)	*n* = 314 (20.46)	
Some college or AA degree	*n* = 5252 (31.50)	*n* = 404 (31.69)	
College graduate or above	*n* = 4026 (28.71)	*n* = 358 (32.02) ^c^	
Age (in years)	45.36 (0.28)	61.75 (0.51)	**< 0.001** ^**d**^
Body mass index (in kg/m^2^)	*n* = 28.85 (0.08)	*n* = 29.20 (0.26)	0.210

Categorical variables are shown as n (weighted %), continuous variables as means (standard error)

^a^ The *p*-value is based on a design-based Rao-Scott *F*-Test and tests for a potential association between cancer survivor status and the respective demographic, anthropometric or clinical characteristics (available for categorical variables only)

^b^ includes 12th grade without diploma

^c^ indicates significant differences in the proportions

^d^ indicates significant differences in the means

Energy intake in cancer survivors was significantly lower as compared to the general population (1881.02 kcal/d vs 2201.78 kcal/d). Table 2 shows daily energy-adjusted nutrient intake (in g/1000 kcal or mg/1000 kcal, respectively) for both groups.

**Table 2:** Nutrient intake – a comparison between cancer survivors and the general population

	General population *n* = 17,969	Cancer Survivors: *n* = 1444	*p*
Energy intake (kcal/day)	2201.78 (10.67)	1881.02 (28.90)	**< 0.001**
Protein intake (g/1000 kcal)	39.19 (0.16)	39.32 (0.49)	0.796
Carbohydrate intake (g/1000 kcal)	121.87 (0.38)	121.50 (1.05)	0.712
Fat (g/1000 kcal)	37.23 (0.14)	38.13 (0.33)	**0.016**
Magnesium intake (mg/1000 kcal)	146.60 (0.81)	153.88 (2.02)	**< 0.001**
Potassium intake (mg/1000 kcal)	1307.81 (6.69)	1422.01 (17.81)	**< 0.001**
Calcium intake (mg/1000 kcal)	464.54 (2.44)	493.46 (7.88)	**< 0.001**
Phosphorus intake (mg/1000 kcal)	656.18 (2.13)	670.76 (7.52)	0.051
Fiber intake (g/1000 kcal)	8.16 (0.07)	8.75 (0.19)	**0.001**

Data shown as mean and standard error in parenthesis. A *p*-value < 0.05 indicates significant differences between both groups

Energy adjusted intake of fat, fiber, potassium, magnesium, and calcium was significantly higher in cancer survivors. Energy-adjusted phosphorus intake also tended to be higher in this group (670.76 mg/1000 kcal vs 656.18 mg/1000 kcal), however, the difference was not statistically significant.

**Fig. 1:**
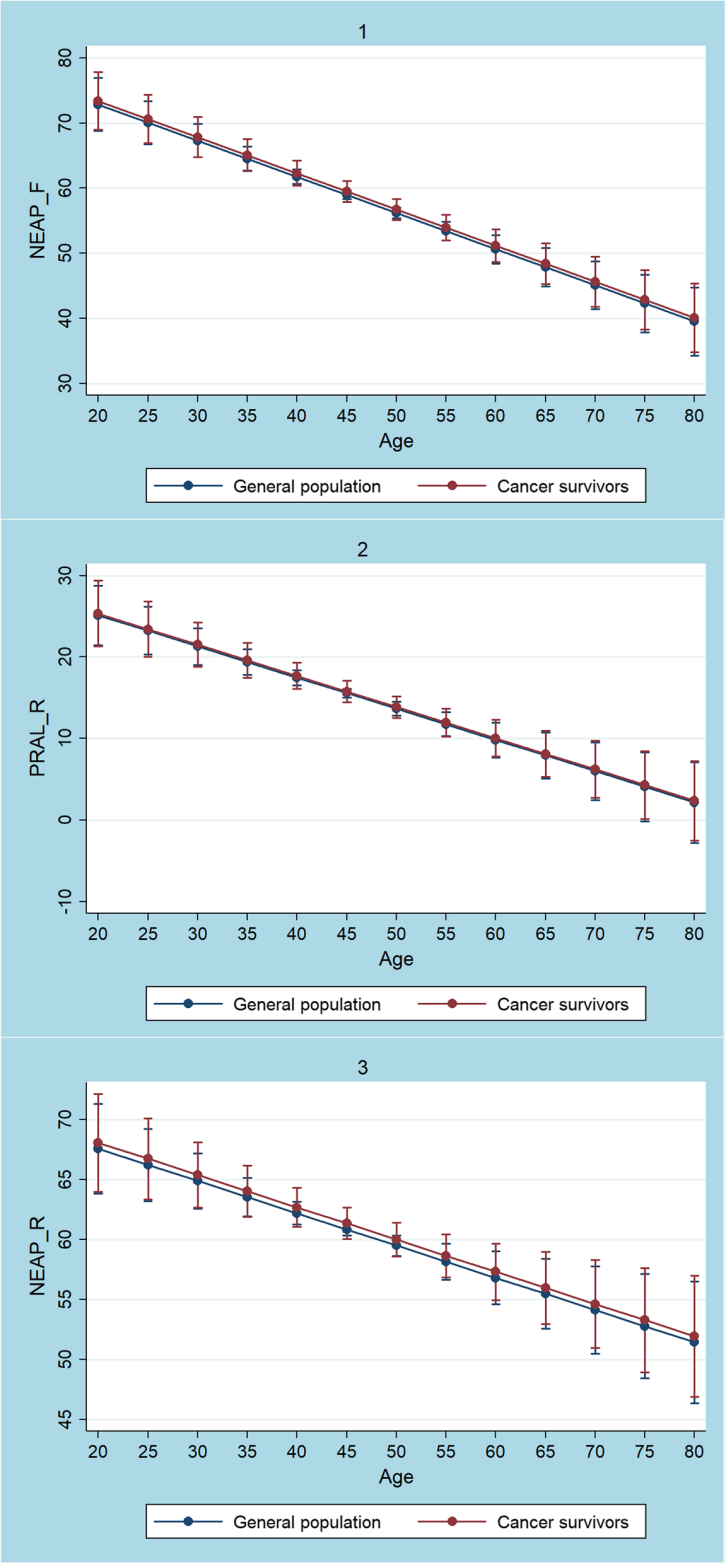
Plots of marginal predicted values for NEAP_F_ (1), PRAL_R_ (2) and NEAP_R_ (3) based on the employed regression models in cancer survivors and in the general population

We ran multiple linear regression analyses to predict DAL scores (NEAP_F_, PRAL_R_, NEAP_R_) from gender, age, race/ethnicity, total energy intake, BMI, and cancer survivor status (which indirectly defined nutrient intake as shown in Table 2). After adjusting for covariates, we found no significant associations between being a self-identified cancer survivor and all 3 DAL scores (NEAP_F_, PRAL_R_, NEAP_R_). Figure 1 displays marginal predicted values of NEAP_F_, PRAL_R_ and NEAP_R_ in cancers survivors at all possible increments of 5 units in age. DAL scores tended to be higher in cancer survivors, however, the differences were not statistically significant. DAL scores declined with higher age but remained positive throughout (e.g. PRAL_R_ > 0 mEq/d), indicating an acidifying potential. Whether cancer survivors modified their diet subsequent to their cancer diagnosis was not ascertainable from out data.

**Fig. 2:**
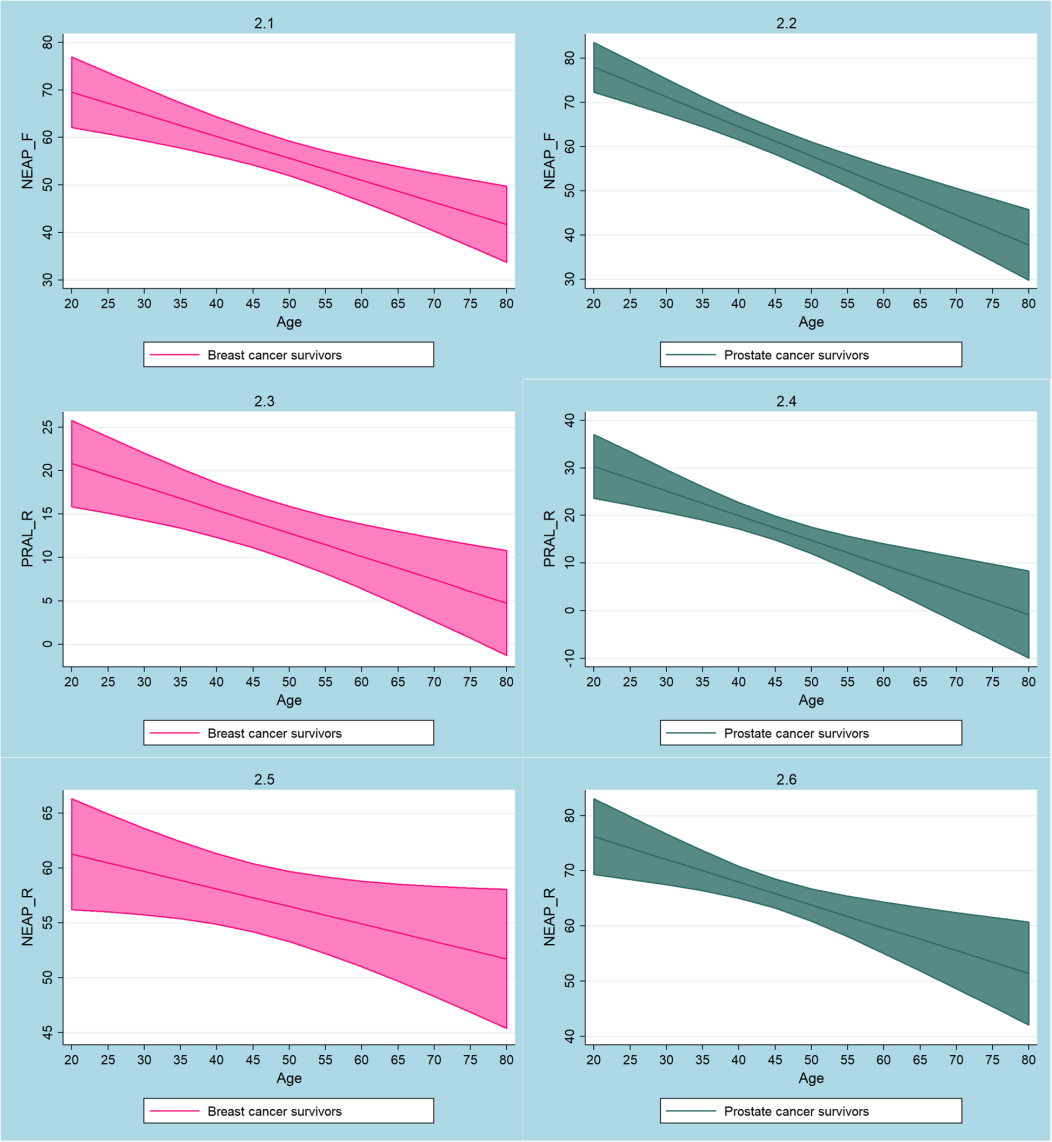
Plots of marginal predicted values (and confidence intervals) for NEAP_F_ (2.1 and 2.2), PRAL_R_ (2.3 and 2.4) and NEAP_R_ (2.5 and 2.6) based on the employed regression models in breast cancer survivors (pink) and prostate cancer survivors (emerald)

Marginal predicted values of NEAP_F_, PRAL_R_ and NEAP_R_ in breast cancer and prostate cancer survivors are shown in Fig. 2. Again, all 3 DAL scores tended to decline with higher age but remained positive throughout. DAL scores were not significantly higher in breast- and prostate cancer survivors as compared to the general population after adjusting for covariates.

Table 3 shows the multivariate linear regression models to predict DAL scores (NEAP_F_, PRAL_R_, and NEAP_R_) from gender, age, race, total energy intake, BMI, and cancer survivor status.

**Table 3:** Multivariate linear regression models to predict DAL scores from gender, age, race, total energy intake, BMI, and cancer survivor status

	NEAP_F_	*p*-value	PRAL_R_	*p*-value	NEAP_R_	*p*-value
Gender						
Male	–		–		–	
Female	−4.53 (−5.40 – (−3.66))	< 0.001	−3.69 (−4.55 – (−2.83))	< 0.001	−9.49 (−10.34 – (−8.64))	< 0.001
Ethnicity						
Mexican American	–		–		–	
Other Hispanic	0.68 (−1.14–2.49)	0.460	−3.09 (−4.82 – (− 1.35))	0.001	− 2.58 (− 4.32 – (−0.84))	0.004
Non-Hispanic White	−2.14 (− 3.60 – (−0.67))	0.005	−3.96 (− 5.39 – (− 2.54))	< 0.001	− 1.07 (− 2.54–0.39)	0.148
Non-Hispanic Black	6.12 (4.66–7.58)	< 0.001	− 0.78 (− 2.19–0.62)	0.273	1.75 (0.31–3.20)	0.018
Other Race - Including Multi-Racial	−0.39 (− 2.51–1.73)	0.714	− 3.06 (− 5.11 – (− 1.01))	0.004	− 2.24 (− 4.28 – (− 0.20))	0.032
Cancer Survivor						
No	–		–		–	
Yes	0.54 (−1.07–2.14)	0.506	0.21 (− 1.19–1.60)	0.768	0.50 (− 0.92–1.92)	0.486
Body mass index (in kg/m^2^)	0.34 (0.26–0.42)	< 0.001	0.28 (0.22–0.35)	< 0.001	0.91 (0.84–0.97)	< 0.001
Total energy intake (in kcal/d)	0.0014 (0.001–0.0018)	< 0.001	0.0098 (0.0089–0.0105)	< 0.001	0.010 (0.092–0.0108)	< 0.001
Age (in years)	−0.56 (− 0.71 – (− 0.40)	< 0.001	−0.38 (− 0.52 – (− 0.24)	< 0.001	−0.27 (− 0.41 – (− 0.12)	< 0.001
Age squared (in years)	0.0025 (0.001–0.004)	< 0.001	0.0019 (0.0005–0.0032)	0.009	0.0003 (− 0.0011–0.0018)	0.635

Coefficients are displayed with their 95% confidence intervals and *p*-values. The symbol “- “indicates the reference category

Apart from cancer survivor status, all entered variables added statistically significantly to the prediction.

## Discussion

The present study sought to quantify DAL in U.S. cancer survivors. All examined acid load scores (NEAP_F_, PRAL_R_, NEAP_R_) were higher in cancer survivors (compared to the general population) after adjustment for confounders. The differences, however, were not statistically significant. DAL scores were positive throughout (> 0 mEq/d), and suggested that cancer survivors in general consumed an acidifying diet that has been associated with adverse effects in the existing studies in cancer survivors [27–30].

Notably, it is important to highlight that we did not perform a case-control study, and that we did not match participants on any characteristics (which poses a bias-susceptible technical challenge because our data stem from a complex multistage, stratified, clustered and probability sampling design). Moreover, the employed NHANES data did not allow us to examine whether cancer survivors modified their diet after receiving a cancer diagnosis.

It is now widely accepted that a change in unhealthy lifestyle behaviors (e.g. smoking cessation, improving diet quality and increasing physical activity) in cancer survivors may help to reduce cancer treatment sequelae, and also reduces the risk for other common diseases such as cardiovascular disorders and obesity [55]. Several studies reported significant and long-term changes in dietary intake in cancer survivors [56–58].

A large Chinese trial found a substantial reduction in the consumption of red meat (*p* < 0.001), processed meat (*p* < 0.001), poultry (*p* < 0.001), and dairy products (*p* < 0.001) in cancer survivors at 18-months post-diagnosis [56]. A French study using data from the NutriNet-Santé cohort observed comparable trends, and reported a decrease in total energy intake (− 377.2 ± 243.5 kcal/d) and protein intake (− 17.4 ± 12.5 g/d) in cancer survivors [57]. The latter was also observed in a study from Malaysia examining dietary intake 2 years after a diagnosis of breast cancer [58]. An Australian study with more than 500 cancer survivors attending the Sydney Cancer Survivorship Center reported that the majority of survivors modified their diet after their cancer diagnosis [59].

We could not examine these associations due to the cross-sectional nature of our data and due to the lack of information on cancer-related comorbidities. As such, our data serve as a mere description of DAL scores in cancer survivors. The comparison to the general population (denying any prior cancer diagnosis) is therefore difficult, as well. We may only speculate why total energy intake was lower in cancer survivors and the same applies for the slightly higher energy-adjusted fat intake in the cancer survivor group (38.13 vs 37.23 g/1000 kcal, *p* = 0.016). It is conceivable that the lower total energy intake is related to the higher age of cancer survivors (61.75 vs 45.36 years). Yet, the cross-sectional nature of our data does not allow for causal attributions. Other scenarios (e.g. unintended therapy side-effects and sequelae including lack of appetite and nausea that lead to a reduced food intake) are also possible. Again, we cannot not prove or reject these hypotheses based on the type of our employed data.

As described in detail earlier, the major aim of this study was acid load quantification in the cancer survivor group. It is possible that cancer survivors in our cohort also modified their diet post diagnosis, but this remains subject to speculation. Although it is conceivable that this was the case (in light of the existing literature on dietary modifications in cancer survivors [56–59]), we may not prove it. Nonetheless, DAL scores were positive throughout in our cohort of cancer survivors, suggesting an acidifying diet (e.g. NEAP_F_ ranging from 50 mEq/d to 60 mEq/d in cancer survivors aged 50 to 60) [60, 61]. This was the case in all 3 models examining cancer survivors in general (Fig. 1), and breast and prostate cancer survivors in particular (Fig. 2).

DAL scores in the range of 50 mEq/d to 60 mEq/d have been associated with adverse health effects and repercussions in analyses using data from the WHEL study in breast cancer survivors [27–30]. Comparable PRAL and NEAP scores have also been associated with an increased mortality in breast cancer survivors [27], and may contribute to reduced overall physical health [28]. Moreover, Wu et al. also reported associations between DAL and elevated plasma CRP and HbA1c levels in breast cancer survivors [29]. Both are important risk factors associated with cancer recurrence and comorbidities in in this particular group [62, 63]. A high DAL may contribute to systemic inflammation and hyperglycemia in cancer survivors [29], which, in turn, has been associated with a worse prognosis.

It is now widely accepted that a high DAL is associated with numerous health repercussions in both healthy and sick individuals [64, 65]. A high acid load decreases blood pH towards the lower end of the normal physiological range and induces low-grade mild metabolic acidosis that causes tissue damage and inflammation [4, 29, 64]. The latter is of particular concern in cancer patients, who have a reduced capacity to adjust their acid-base balance [66]. Local inflammation subsequent to an acidic microenvironment may initiate genomic instability on normal cells through the activation of cytokines, which may stimulate tumor invasion and metastases [15, 16, 67]. The combined evidence from basic research [13–16] and epidemiological investigations [29, 30, 32, 33] warrants consideration, and additional trials should investigate potential adverse effect of a high DAL in cancer survivors.

Our study provides evidence that a high diet-dependent acid load is common in cancer survivors in the NHANES and emphasizes the need for additional research in this area of current oncological interest. It is of utmost importance to highlight that DAL is an easily modifiable risk factor, as dietary interventions promoting more plant-based diets were shown to reduce acid load from diet [5, 68, 69]. The fact that our results still revealed a higher acid load in cancer survivors (who most likely modified and improved their diet after diagnosis) as compared to the general population reinforces this call.

### Strengths and limitations

This study has several strengths and limitations that warrant further discussion. To the best of our knowledge, it is one of the first studies to quantify DAL in a large and nationally-representative cohort of cancer survivors (NHANES). As such, we may have built the foundation for additional research in the future in this field. Although we did not match participants (e.g. “cases and controls”) due to the specific nature of our data (complex multistage, stratified, clustered data), we used state-of-the-art multivariate linear regression techniques to adjust for confounders (such as age and total energy intake). An additional asset of our studies is the fact the we employed 3 different DAL scores (NEAP_F_, PRAL_R_, NEAP_R_), and did not restrict our analysis to PRAL_R_ and NEAP_F_, which is often the case in epidemiological research. As such, our study also includes a marker based on anthropometric data (NEAP_R_).

Weaknesses include the missing cancer-specific parameters (e.g. duration since cancer diagnosis, the exact cancer stage, treatments received, hormonal status for breast cancer, etc.). This information would have been valuable to allow for a more detailed description of cancer cases. Discussing these parameters in the context of acid load scores would have certainly enriched our study but unfortunately they were not available in the NHANES. On the other hand, none of these cancer-specific parameters is directly related to the DAL calculations, which is based on nutrients and anthropometric data. Moreover, cancer status was self-reported (see methods) which could theoretically lead to bias. The lack of matching methods in the sense of a case-control study could also be interpreted as a weakness. Yet again, the major aim of this study was to quantify DAL in cancer survivors to gain a first overall impression, and to investigate whether elevated DAL scores were eventually a topic in cancer survivors or not. Our results suggest that additional trials in this field are urgently warranted, particularly with regard to specific cancers. Future studies should also examine additional associations between specific clinical outcomes and an elevated DAL.

## Conclusions

The present study investigated DAL in U.S. cancer survivors from the NHANES and revealed a higher (yet non-significant) diet-dependent acid load in this cohort. DAL scores > 0 mEq/d suggested an acidifying diet in cancer survivors. This warrants attention, as comparable acid load scores have been associated with adverse health outcomes in previous studies in breast cancer survivors.

## Data Availability

Data is publicly available online (https://wwwn.cdc.gov/nchs/nhanes/Default.aspx). The datasets used and analyzed during the current study are available from the corresponding author on reasonable request.

## Abbreviations

AMPM: Automated Multiple Pass Method
BMI: Body Mass Index
CI: Confidence Interval
CRP: C Reactive Protein
DAL: Dietary Acid Load
HbA1c: Hemoglobin A1c
NCHS: National Center for Health Statistics
NEAP: Net Endogenous Acid Production
NHANES: National Health and Nutrition Examination Survey
PRAL: Potential Renal Acid Load
USDA: U.S. Department of Agriculture
WHEL: Women’s Healthy Eating and Living

## References

[1] Banerjee T, Crews DC, Wesson DE, Tilea A, Saran R, Rios Burrows N (2014). Dietary acid load and chronic kidney disease among adults in the United States. BMC Nephrol.

[2] Kahleova H, McCann J, Alwarith J, Rembert E, Tura A, Holubkov R (2021). A plant-based diet in overweight adults in a 16-week randomized clinical trial: the role of dietary acid load. Clin Nutr ESPEN.

[3] Vormann J, Remer T (2008). Dietary, metabolic, physiologic, and disease-related aspects of acid-base balance: foreword to the contributions of the second international acid-base symposium. J Nutr.

[4] Williams RS, Kozan P, Samocha-Bonet D (2016). The role of dietary acid load and mild metabolic acidosis in insulin resistance in humans. Biochimie..

[5] Müller A, Zimmermann-Klemd AM, Lederer A-K, Hannibal L, Kowarschik S, Huber R (2021). A vegan diet is associated with a significant reduction in dietary acid load: post hoc analysis of a randomized controlled trial in healthy individuals. Int J Environ Res Public Health.

[6] Remer T, Manz F (1995). Potential renal acid load of foods and its influence on urine pH. J Am Diet Assoc.

[7] Adeva MM, Souto G (2011). Diet-induced metabolic acidosis. Clin Nutr.

[8] Storz MA, Ronco AL. Reduced dietary acid load in U.S. vegetarian adults: Results from the National Health and Nutrition Examination Survey. Food Science & Nutrition. [cited 2022 Apr 12];n/a(n/a). Available from: https://onlinelibrary.wiley.com/doi/abs/10.1002/fsn3.2825 10.1002/fsn3.2825 PMC9179160 35702310

[9] Lee KW, Shin D (2020). Positive association between dietary acid load and future insulin resistance risk: findings from the Korean genome and epidemiology study. Nutr J.

[10] Scialla JJ, Appel LJ, Astor BC, Miller ER, Beddhu S, Woodward M (2011). Estimated net endogenous acid production and serum bicarbonate in African Americans with chronic kidney disease. Clin J Am Soc Nephrol.

[11] Hejazi E, Emamat H, Sharafkhah M, Saidpour A, Poustchi H, Sepanlou S, et al. Dietary acid load and mortality from all causes, CVD and cancer: results from the Golestan cohort study. Br J Nutr. 2021:1–7. 10.1017/S0007114521003135 34392847

[12] Park Y-MM, Steck SE, Fung TT, Merchant AT, Elizabeth Hodgson M, Keller JA (2019). Higher diet-dependent acid load is associated with risk of breast cancer: findings from the sister study. Int J Cancer.

[13] Robey IF (2012). Examining the relationship between diet-induced acidosis and cancer. Nutr Metab.

[14] Gillies RJ, Verduzco D, Gatenby RA (2012). Evolutionary dynamics of carcinogenesis and why targeted therapy does not work. Nat Rev Cancer.

[15] Gillies RJ, Pilot C, Marunaka Y, Fais S (2019). Targeting acidity in cancer and diabetes. Biochim Biophys Acta Rev Cancer.

[16] Moellering RE, Black KC, Krishnamurty C, Baggett BK, Stafford P, Rain M (2008). Acid treatment of melanoma cells selects for invasive phenotypes. Clin Exp Metastasis.

[17] Keramati M, Kheirouri S, Musazadeh V, Alizadeh M. Association of high dietary acid load with the risk of cancer: a systematic review and meta-analysis of observational studies. Front Nutr. 2022;9 [cited 2022 Apr 12]. Available from: https://www.frontiersin.org/article/10.3389/fnut.2022.816797. 10.3389/fnut.2022.816797 PMC8997294 35419387

[18] Dalamaga M, Diakopoulos KN, Mantzoros CS (2012). The role of adiponectin in cancer: a review of current evidence. Endocr Rev.

[19] Hua H, Kong Q, Yin J, Zhang J, Jiang Y (2020). Insulin-like growth factor receptor signaling in tumorigenesis and drug resistance: a challenge for cancer therapy. J Hematol Oncol.

[20] Ronco AL, Storz MA, Martínez-López W, Calderón JM, Golomar W (2021). High dietary acid load is associated with prostate cancer risk: an epidemiological study. WCRJ.

[21] Shi L-W, Wu Y-L, Hu J-J, Yang P-F, Sun W-P, Gao J (2021). Dietary acid load and the risk of pancreatic cancer: a prospective cohort study. Cancer Epidemiol Biomark Prev.

[22] Ronco A, Calderón J, Mendoza B (2020). Dietary acid load and breast cancer risk: a case-control study in Uruguay. Eur J Cancer.

[23] Ronco AL, Martínez-López W, Calderón JM, Golomar W (2021). Dietary acid load and lung cancer risk: a case-control study in men. Cancer Treat Res Commun.

[24] Jafari Nasab S, Rafiee P, Bahrami A, Rezaeimanesh N, Rashidkhani B, Sohrab G (2021). Diet-dependent acid load and the risk of colorectal cancer and adenoma: a case-control study. Public Health Nutr.

[25] Ronco AL, Martínez-López W, Calderón JM, Mendoza BA (2020). Dietary acid load and colorectal cancer risk: a case-control study. World Cancer Res J.

[26] Milajerdi A, Shayanfar M, Benisi-Kohansal S, Mohammad-Shirazi M, Sharifi G, Tabibi H (2021). A case-control study on dietary acid load in relation to glioma. Nutr Cancer.

[27] Wu T, Hsu F-C, Pierce JP (2020). Increased acid-producing diet and past smoking intensity are associated with worse prognoses among breast cancer survivors: a prospective cohort study. J Clin Med.

[28] Tessou KD, Lemus H, Hsu F-C, Pierce J, Hong S, Brown L (2021). Independent and joint impacts of acid-producing diets and depression on physical health among breast cancer survivors. Nutrients..

[29] Wu T, Seaver P, Lemus H, Hollenbach K, Wang E, Pierce JP (2019). Associations between dietary acid load and biomarkers of inflammation and hyperglycemia in breast cancer survivors. Nutrients..

[30] Chronister BNC, Wu T, Santella RM, Neugut AI, Wolff MS, Chen J (2022). Dietary acid load, serum polychlorinated biphenyl levels, and mortality following breast cancer in the Long Island breast cancer study project. Int J Environ Res Public Health.

[31] Wu T, Hsu F-C, Pierce JP (2020). Acid-producing diet and depressive symptoms among breast cancer survivors: a longitudinal study. Cancers (Basel).

[32] NHANES - About the National Health and Nutrition Examination Survey. 2020 [cited 2022 Feb 11]. Available from: https://www.cdc.gov/nchs/nhanes/about_nhanes.htm

[33] NHANES - Participants - About. 2021 [cited 2022 Feb 11]. Available from: https://www.cdc.gov/nchs/nhanes/participant/participant-about.htm

[34] Albanes D, Blair A, Taylor PR (1989). Physical activity and risk of cancer in the NHANES I population. Am J Public Health.

[35] Lynch BM, Dunstan DW, Healy GN, Winkler E, Eakin E, Owen N (2010). Objectively measured physical activity and sedentary time of breast cancer survivors, and associations with adiposity: findings from NHANES (2003–2006). Cancer Causes Control.

[36] Marinac CR, Natarajan L, Sears DD, Gallo LC, Hartman SJ, Arredondo E (2015). Prolonged nightly fasting and breast cancer risk: findings from NHANES (2009–2010). Cancer Epidemiol Biomark Prev.

[37] Deshmukh AA, Shirvani SM, Likhacheva A, Chhatwal J, Chiao EY, Sonawane K. The association between dietary quality and overall and cancer-specific mortality among cancer survivors, NHANES III. JNCI Cancer Spectr. 2018;2(2) [cited 2022 Feb 11]. Available from: https://academic.oup.com/jncics/article/2/2/pky022/5026131. 10.1093/jncics/pky022 PMC5989369 29905226

[38] Su LJ, Arab L (2001). Nutritional status of folate and colon cancer risk: evidence from NHANES I epidemiologic follow-up study. Ann Epidemiol.

[39] Petrova D, Catena A, Rodríguez-Barranco M, Redondo-Sánchez D, Bayo-Lozano E, Garcia-Retamero R (2021). Physical comorbidities and depression in recent and long-term adult cancer survivors: NHANES 2007–2018. Cancers..

[40] Karavasiloglou N, Pestoni G, Faeh D, Rohrmann S (2019). Post-diagnostic diet quality and mortality in females with self-reported history of breast or gynecological cancers: results from the third National Health and Nutrition Examination Survey (NHANES III). Nutrients..

[41] NHANES - NCHS Research Ethics Review Board Approval. 2021 [cited 2022 Feb 11]. Available from: https://www.cdc.gov/nchs/nhanes/irba98.htm

[42] NHANES 2007–2008: Dietary Interview - Individual Foods, First Day Data Documentation, Codebook, and Frequencies. [cited 2022 Apr 13]. Available from: https://wwwn.cdc.gov/Nchs/Nhanes/2007-2008/DR1IFF_E.htm

[43] NHANES - Measuring Guides. 2019 [cited 2022 Apr 13]. Available from: https://www.cdc.gov/nchs/nhanes/measuring_guides_dri/measuringguides.htm

[44] AMPM—USDA Automated Multiple-Pass Method: USDA ARS. Available online: https://www.ars.usda.gov/northeast-area/beltsville-md-bhnrc/beltsville-human-nutrition-research-center/food-surveys-research-group/docs/ampm-usda-automatedmultiple-pass-method/ (Accessed on 31 Jan 2022).

[45] Centers for Disease Control and Prevention, National Center for Health Statistics (2008). National Health and Nutrition Examination Survey MEC in-person dietary interviewers procedure manual.

[46] Frassetto LA, Todd KM, Morris RC, Sebastian A (1998). Estimation of net endogenous noncarbonic acid production in humans from diet potassium and protein contents. Am J Clin Nutr.

[47] Remer T, Dimitriou T, Manz F (2003). Dietary potential renal acid load and renal net acid excretion in healthy, free-living children and adolescents. Am J Clin Nutr.

[48] Parmenter BH, Slater GJ, Frassetto LA (2017). Accuracy and precision of estimation equations to predict net endogenous acid excretion using the Australian food database. Nutr Diet.

[49] Parmenter BH, Dymock M, Banerjee T, Sebastian A, Slater GJ, Frassetto LA (2020). Performance of predictive equations and biochemical measures quantifying net endogenous acid production and the potential renal acid load. Kidney Int Rep.

[50] Parker JD, Talih M, Malec DJ, Beresovsky V, Carroll M, Gonzalez JF, et al. National Center for Health Statistics data presentation standards for proportions. Vital Health Stat 2. 2017;(175):1–22. 30248016

[51] Ward BW (2019). kg_nchs: a command for Korn–Graubard confidence intervals and National Center for Health Statistics’ data presentation standards for proportions. Stata J.

[52] Alam I, Alam I, Paracha PI, Pawelec G (2012). Higher estimates of daily dietary net endogenous acid production (NEAP) in the elderly as compared to the young in a healthy, free-living elderly population of Pakistan. CIA..

[53] Lemann J (1999). Relationship between urinary calcium and net acid excretion as determined by dietary protein and potassium: a review. Nephron..

[54] Gannon RHT, Millward DJ, Brown JE, Macdonald HM, Lovell DP, Frassetto LA (2008). Estimates of daily net endogenous acid production in the elderly UK population: analysis of the National Diet and Nutrition Survey (NDNS) of British adults aged 65 years and over. Br J Nutr.

[55] Pinto BM, Eakin E, Maruyama NC (2000). Health behavior changes after a cancer diagnosis: what do we know and where do we go from here?. Ann Behav Med.

[56] Lei Y-Y, Ho SC, Cheng A, Kwok C, Cheung KL, He Y-Q (2018). Dietary changes in the first 3 years after breast cancer diagnosis: a prospective Chinese breast cancer cohort study. Cancer Manag Res.

[57] Fassier P, Zelek L, Lécuyer L, Bachmann P, Touillaud M, Druesne-Pecollo N (2017). Modifications in dietary and alcohol intakes between before and after cancer diagnosis: results from the prospective population-based NutriNet-Santé cohort. Int J Cancer.

[58] Shaharudin SH, Sulaiman S, Shahril MR, Emran NA, Akmal SN (2013). Dietary changes among breast cancer patients in Malaysia. Cancer Nurs.

[59] Tan SY, Wong HY, Vardy JL (2021). Do cancer survivors change their diet after cancer diagnosis?. Support Care Cancer.

[60] Remer T, Berkemeyer S, Rylander R, Vormann J (2007). Muscularity and adiposity in addition to net acid excretion as predictors of 24-h urinary pH in young adults and elderly. Eur J Clin Nutr.

[61] Lemann JrJ (1999). Relationship between urinary calcium and net acid excretion as determined by dietary protein and potassium: a review. NEF..

[62] McAndrew NP, Bottalico L, Mesaros C, Blair IA, Tsao PY, Rosado JM (2021). Effects of systemic inflammation on relapse in early breast cancer. Npj Breast Cancer.

[63] Ayoub NM, Jaradat SK, Alhusban A, Tahaineh L (2020). <p>glycosylated hemoglobin A1c is associated with anthropometric measurements and tumor characteristics in breast cancer patients. IJWH..

[64] Osuna-Padilla IA, Leal-Escobar G, Garza-García CA, Rodríguez-Castellanos FE (2019). Dietary acid load: mechanisms and evidence of its health repercussions. Nefrologia (Engl Ed).

[65] Abshirini M, Bagheri F, Mahaki B, Siassi F, Koohdani F, Safabakhsh M (2019). The dietary acid load is higher in subjects with prediabetes who are at greater risk of diabetes: a case–control study. Diabetol Metab Syndr.

[66] Sia P, Plumb TJ, Fillaus JA (2013). Type B lactic acidosis associated with multiple myeloma. Am J Kidney Dis.

[67] Estrella V, Chen T, Lloyd M, Wojtkowiak J, Cornnell HH, Ibrahim-Hashim A (2013). Acidity generated by the tumor microenvironment drives local invasion. Cancer Res.

[68] Cosgrove K, Johnston CS (2017). Examining the impact of adherence to a vegan diet on acid-base balance in healthy adults. Plant Foods Hum Nutr.

[69] Knurick JR, Johnston CS, Wherry SJ, Aguayo I (2015). Comparison of correlates of bone mineral density in individuals adhering to lacto-ovo, vegan, or omnivore diets: a cross-sectional investigation. Nutrients..

